# Whole Exome Sequencing in Patients with the Cuticular Drusen Subtype of Age-Related Macular Degeneration

**DOI:** 10.1371/journal.pone.0152047

**Published:** 2016-03-23

**Authors:** Maheswara R. Duvvari, Johannes P. H. van de Ven, Maartje J. Geerlings, Nicole T. M. Saksens, Bjorn Bakker, Arjen Henkes, Kornelia Neveling, Marisol del Rosario, Dineke Westra, Lambertus P. W. J. van den Heuvel, Tina Schick, Sascha Fauser, Camiel J. F. Boon, Carel B. Hoyng, Eiko K. de Jong, Anneke I. den Hollander

**Affiliations:** 1 Department of Ophthalmology, Radboud University Medical Centre, Nijmegen, the Netherlands; 2 Department of Human Genetics, Radboud University Medical Centre, Nijmegen, the Netherlands; 3 Department of Pediatric Nephrology, Radboud University Medical Centre, Nijmegen, the Netherlands; 4 Department of Ophthalmology, University Hospital of Cologne, Cologne, Germany; 5 Department of Ophthalmology, Leiden University Medical Center, Leiden, the Netherlands; University of Florida, UNITED STATES

## Abstract

Age-related macular degeneration (AMD) is the leading cause of irreversible blindness in elderly people worldwide. Cuticular drusen (CD) is a clinical subtype of AMD, which typically displays an earlier age at onset, and has a strong genetic component. Genetic studies support a role for rare sequence variants in CD susceptibility, and rare sequence variants in the *CFH* gene have been identified in 8.8% of CD cases. To further explore the role of rare variants in CD, we performed whole exome sequencing (WES) in 14 affected members of six families and 12 sporadic cases with CD. We detected rare sequence variants in *CFH* and *FBLN5*, which previously were shown to harbor rare variants in patients with CD. In addition, we detected heterozygous rare sequence variants in several genes encoding components of the extracellular matrix (ECM), including *FBLN1*, *FBLN3*/*EFEMP1*, *FBLN5*, *FBLN6*/*HMCN1*, *FBN2*, and *COL15A1*. Two rare pathogenic variants were identified in the *COL15A1* gene: one in a sporadic case and another was found to segregate in a family with six affected individuals with CD. In addition, two rare pathogenic variants were identified in the *FGL1* gene in three unrelated CD cases. These findings suggest that alterations in the ECM and in the coagulation pathway may play a role in the pathogenesis of CD. The identified candidate genes require further analyses in larger cohorts to confirm their role in the CD subtype of AMD. No evidence was found of rare sequence variants in a single gene that segregate with CD in the six families, suggesting that the disease is genetically heterogeneous.

## Introduction

Age-related macular degeneration (AMD, OMIM 603075) is a leading cause of visual impairment and affects 8.7% of elderly people worldwide [1]. An early pathological symptom is the formation of drusen in the macula, the central region of the retina that is necessary for sharp and central vision. AMD is a clinically heterogeneous disorder that displays a broad spectrum of clinical appearances [2–5]. Cuticular drusen (CD, OMIM 126700) is a clinical subtype of AMD, characterized by the presence of at least 50 small (25–75μm) uniformly sized hyperfluorescent drusen, scattered primarily in the macular region on fluorescein angiography (FA) [6]. There is evidence that CD has a strong genetic component; CD often occurs in families and it presents clinically at an earlier age at onset than AMD [7,8]. Also, the most commonly associated environmental factor, smoking, shows a weaker association with CD than with the non-CD type AMD [9]. Genetic studies further support that CD has a strong genetic component by showing significant associations of CD with common variants (minor allele frequency [MAF] ≥ 1%) in the *CFH* (OMIM 134370), *ARMS2* (OMIM 611313) *C2* (OMIM 613927)/*CFB* (OMIM 138470), *C3* (OMIM 120700), and *APOE* (OMIM 107741) genes [7,9]. Moreover, heterozygous mutations in *CFH* segregate in multiplex CD families [10,11] and a highly penetrant AMD risk variant, p.Arg1210Cys in *CFH*, was identified in two CD cases [12]. Furthermore, we recently demonstrated that 8.8% of CD cases harbor rare sequence variants (MAF ≤ 1%) in *CFH* [12]. This evidence supports a strong genetic component in CD, but additional genetic factors that contribute to CD susceptibility are yet to be discovered. Whole exome sequencing (WES) selectively sequences all protein-coding regions of the genome, known as the exome. Protein-coding regions are collectively approximately 30 megabases in size, spread across 180,000 exons that constitute 1% of the human genome [13], and are estimated to harbor 85% of disease-causing mutations [14]. Therefore, WES offers an unprecedented opportunity to study the role of rare sequence variants in protein-coding regions in complex diseases. In the present study, we sought to determine the role of rare sequence variants in CD using WES. We performed WES in 14 affected members of six families and 12 sporadic cases with CD. In addition, we conducted segregation analysis for rare sequence variants that were identified by WES, in affected individuals of six families.

## Materials and Methods

### Patients

**Fig 1:**
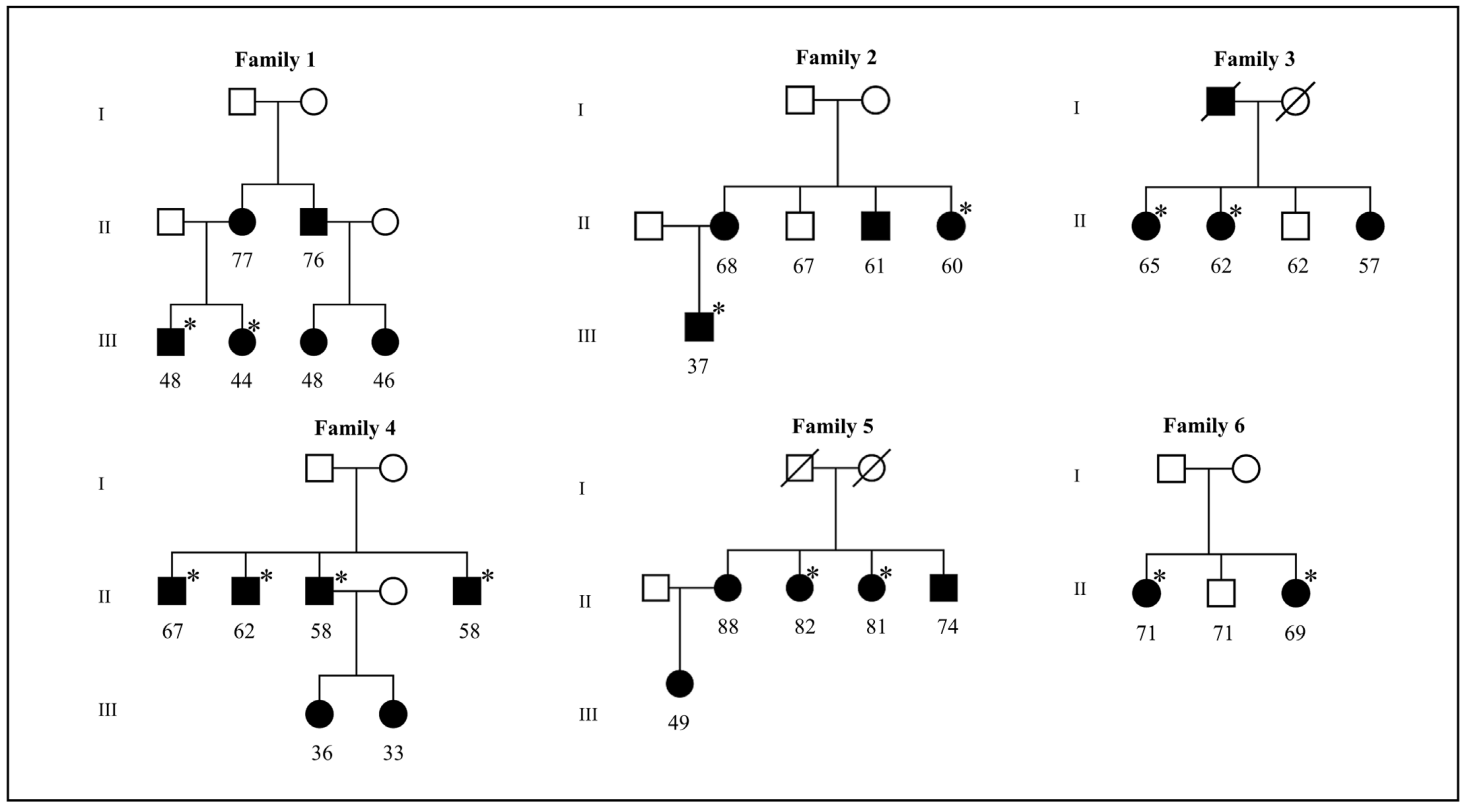
Pedigrees of six cuticular drusen (CD) families in which whole exome sequencing (WES) was performed. Circle and square symbols indicate female and male individuals, respectively. Symbols with slashes indicate deceased individuals. Black and empty symbols indicate affected and unaffected individuals, respectively. Asterisks indicate the family members for whom exome sequencing was performed. The numbers below the symbols indicate the age at participation of family members.

**Fig 2:**
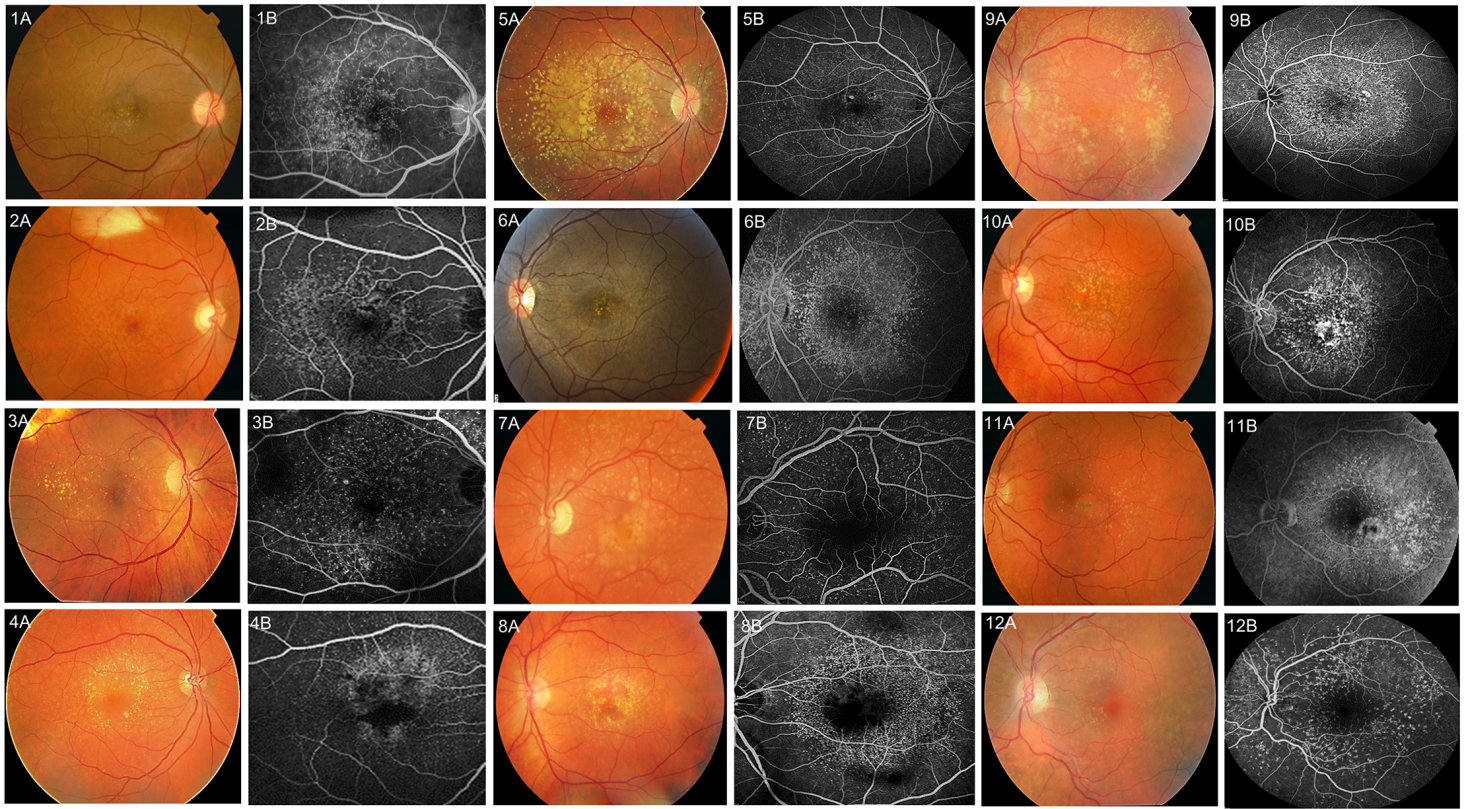
Retinal images of 12 sporadic cuticular drusen (CD) cases for whom exome sequencing (WES) was performed. Panels A and B represent colour fundus photographs (1A-12A) and fluorescein angiograms (FAs) (1B-12B) of 12 cases respectively. For cases 1–5 retinal images of the right eye are shown, whereas for cases 6–12 retinal images of the left eye are shown. The CD phenotype presents with a large number of small and uniformly sized hyperfluorescent drusen on FA.

We performed WES in 14 affected members of six families and 12 sporadic cases with the CD subtype of AMD (Figs 1 and 2). All study participants were selected from the European Genetic Database (EUGENDA) and are of Caucasian descent. All participants underwent extensive retinal examinations, which is described elsewhere in detail [12,15]. CD was diagnosed on the basis of the clinical observation of a symmetrically distributed pattern in both eyes of at least 50 scattered, uniformly-sized, small (25–75μm), and hyperfluorescent drusen on FA in each eye, and with a minimum of 20 drusen located outside the Wisconsin age-related maculopathy grading template [16,17]. The EUGENDA study was approved by the local research ethics committees (Commissie Mensgebonden Onderzoek Regio Arnhem-Nijmegen, the Netherlands, and Ethics Committee of the University Hospital Cologne, Germany). The study adhered to the ARVO statement for the use of human subjects in ophthalmic and vision research, and was performed in accordance with the tenets of the Declaration of Helsinki. Written informed consent was obtained from all participants. Peripheral blood samples were collected from all participants, and genomic DNA was isolated using the Chemagic STAR DNA Blood4k kit (PerkinElmer, Waltham, MA, USA).

### Whole exome sequencing

The exomes of all study participants were captured using the SureSelect Human All Exon kit version 2 (50Mb; Agilent Technologies, Santa Clara, CA, USA) using 3μg of genomic DNA. Subsequently, captured libraries were sequenced on SOLiD4 or 5500xl sequencing platforms (Life Technologies, Carlsbad, CA, USA). Reads were aligned to the reference human genome (NCBI hg19) with SOLiD LifeScope version 2.1 or SOLiD BioScope version 1.3 software (Life Technologies). Single nucleotide variants (SNVs) were called using the high-stringency DiBayes algorithm, and small insertions and deletions were detected using the SOLiD small Indel Tool (Life Technologies). The pathogenicity of missense variants were predicted in silico using Polymorphism Phenotyping version 2 (PolyPhen-2) and Sorting Intolerant from Tolerant (SIFT) tools.

The exomes were analyzed for variants in 289 candidate genes for CD and atypical hemolytic uremic syndrome (aHUS), because of their known allelic overlap [15,18,19]. The candidate genes were selected from known CD genes [5,7], known AMD loci [20], genes known to be involved in monogenic macular degeneration (Retnet), and genes encoding components of the complement system, coagulation system, innate immunity, endothelial cells, and the glomerular basement membrane (S1 Table) (Westra *et al*, in preparation). The exomes of the six families were also analyzed for variants in all genes of the exome that were shared among all affected individuals of all six families.

### Sanger sequencing

Sanger sequencing was performed to validate rare sequence variants identified by WES. In families, confirmed rare sequence variants were analyzed for segregation by Sanger sequencing in available family members. Primers were designed with Primer3Plus software (Primer3Plus). PCR was performed, and amplification products were sequenced using an automated sequencer (BigDye Terminator, version 3, 3730 DNA analyzer; Applied Biosystems, Waltham, MA, USA). Sequencing chromatograms were compared to the reference sequence using ContigExpress (Vector NTI Advance, version 11.0, Life Technologies).

## Results

### Whole exome sequence analysis

WES was performed in 14 affected members of six families and 12 sporadic cases with the CD subtype of AMD. We detected an average of 34,784 sequence variants per exome, with an average coverage of 70-fold. We subsequently focused our analyses on 289 candidate genes (S1 Table) and detected an average of 780 sequence variants in these genes per sporadic case (S2 Table). In the six families, identically annotated sequence variants (referred to as overlapping variants) were selected that were found in all affected individuals of each family. An average of 508 overlapping variants were detected in these candidate genes per family (S2 Table). We next applied rigorous filtering criteria to identify rare, functionally relevant sequence variants in the candidate genes. The sequence variants were selected when they were present on 10 or more (≥10) variant reads, and when they were present in 20 percent or more (≥20%) of the variant reads. We then selected non-synonymous, coding insertion-deletions (indels), and splice acceptor and donor site variants in the candidate genes, and detected an average of 133 variants in each sporadic case, while on average 88 overlapping variants were detected in each family. Finally, we selected rare sequence variants with a minor allele frequency of ≤1% (MAF ≤0.01) in the 1000 Genomes project (1000 Genomes project) and the global MAF listing in the dbSNP database (dbSNP database). These filtering criteria resulted a total of 98 heterozygous rare sequence variants in 12 sporadic cases, with an average of eight rare sequence variants per sporadic case (S2 Table; S3–S14 Tables). A total of nine heterozygous overlapping variants were detected in four families, with an average of two variants in each family. In two families, no overlapping rare variants were identified (S2 Table).

#### Rare variants in sporadic CD cases

We first examined genes that have previously been described to carry rare variants in macular degeneration, for rare sequence variants in 12 sporadic CD cases. In eight sporadic cases, we detected eight heterozygous rare sequence variants in six macular degeneration genes (*CFH*; *FBLN1*, OMIM 135820; *FBLN3/EFEMP1*, OMIM 601548; *FBLN5*, OMIM 604580; *HMCN1*, OMIM 608548; *FBN2*, OMIM 612570) (Table 1). All rare sequence variants in the *CFH* (p.Ala173Gly; p.Gln950His/rs149474608), *FBLN1* (p.His695Arg/rs13268), *EFEMP1* (p.Asp49Ala/rs55849640), *FBLN5* (p.Val126Met/rs61734479), *HMCN1* (p.Lys2559Asn/rs139899015), and *FBN2* (p.Pro326Ser/rs28763954; p.His1381Asn/rs78727187) genes were confirmed by Sanger sequencing. Five of these variants (p.Gln950His (*CFH*), p.His695Arg (*FBLN1*), p.Val126Met (*FBLN5*), p.Lys2559Asn (*HMCN1*), and p.His1381Asn (*FBN2*)) are predicted to be damaging by prediction tools SIFT and PolyPhen2. However, three variants (p.Ala173Gly (*CFH*), p.Asp49Ala (*EFEMP1*), and p.Pro326Ser (*FBN2*)) are predicted to be damaging by one of these prediction tools. Evolutionary conservation of altered bases were predicted using the Phylop program. The variants in the *FBLN1*, *EFEMP1*, *FBLN5*, *HMCN1*, and *FBN2* genes are conserved, whereas the c.518C>G (p.Ala173Gly) and c.2850G>T (p.Gln950His) variants in the *CFH* gene are less conserved (Table 1).

**Table 1:** Rare missense variants identified in known macular degeneration genes in 12 sporadic CD subtype of AMD cases by WES.

Gene	Change in		dbSNP		Prediction algorithms		Conservation	# Cases (Fig 2)
	Nucleotide	Protein	ID	MAF (%)	SIFT	PolyPhen2	Phylop (Base level)	
*CFH*	c.518C>G	p.Ala173Gly	NA	0	Deleterious	Benign	0.3	1 (3AB)
*CFH*	c.2850G>T	p.Gln950His	rs149474608	0.002	Deleterious	Damaging	-0.7	1 (9AB)
*FBLN1*	c.2084A>G	p.His695Arg	rs13268	0.007	Deleterious	Damaging	4.2	1 (2AB)
*FBLN3/EFEMP1*	c.146T>G	p.Asp49Ala	rs55849640	0.0004	Deleterious	Benign	2.3	1 (12AB)
*FBLN5*	c.376C>T	p.Val126Met	rs61734479	0.0008	Deleterious	Damaging	4.0	1 (10AB)
*FBLN6/HMCN1*	c.7677G>C	p.Lys2559Asn	rs139899015	0	Deleterious	Damaging	1.2	1 (8AB)
*FBN2*	c.976G>A	p.Pro326Ser	rs28763954	0.003	Deleterious	Benign	2.7	1 (9AB)
*FBN2*	c.4141G>T	p.His1381Asn	rs78727187	0	Deleterious	Damaging	5.9	1 (7AB)

CD, cuticular drusen; AMD, age-related macular degeneration; WES, whole exome sequencing; MAF, minor allele frequency; SIFT, sorting intolerant from tolerant; PolyPhen2, polymorphism phenotyping; Phylop score (< 0, less conserved; 0, neutral; > 0 conserved; a large score indicates high conservation); NA, not applicable

We next sought for genes that were burdened recurrently with rare sequence variants in at least three sporadic cases. We detected two recurrent candidate genes (*FGL1*, OMIM 605776; *COL15A1*, OMIM 120325) that harbored rare sequence variants in a total of six sporadic cases (Table 2). Sanger sequencing confirmed variants in the *FGL1* (p.Tyr140Phe/rs35431851; p.Trp256Leu/rs2653414), and *COL15A1* (p.Phe851Leu/rs35901514) genes, while a variant in the *COL15A1* (p.Lys1365Ile) gene was not confirmed by Sanger sequencing (S15 Table). Of the confirmed variants, a rare variant (*FGL1*, p.Trp256Leu/rs2653414) is detected recurrently in two sporadic cases. Two variants in *FGL1* (p.Tyr140Phe; p.Trp256Leu) gene are predicted to be damaging by both SIFT and PolyPhen2. The variant in the *COL15A1* gene (p.Phe851Leu) is predicted to be damaging by one of two algorithms. The Phylop program predicted that all three variants in the *FGL1* and *COL15A1* genes are evolutionarily conserved (Table 2).

**Table 2:** Recurrent missense variants identified in two of 289 candidate genes in 12 sporadic CD subtype of AMD cases by WES.

Gene	Change in		dbSNP		Prediction algorithms		Conservation	# Cases (Fig 2)
	Nucleotide	Protein	ID	MAF (%)	SIFT	PolyPhen2	Phylop (Base level)	
*FGL1*	c.419T>A	p.Tyr140Phe	rs35431851	0.007	Deleterious	Damaging	4.6	1 (11AB)
*FGL1*	c.767C>A	p.Trp256Leu	rs2653414	0.009	Deleterious	Damaging	5.1	2 (4AB; 6AB)
*COL15A1*	c.2551T>C	p.Phe851Leu	rs35901514	0.003	Tolerated	Damaging	2.8	1 (2AB)

CD, cuticular drusen; AMD, age-related macular degeneration; WES, whole exome sequencing; MAF, minor allele frequency; SIFT, sorting intolerant from tolerant; PolyPhen2, polymorphism phenotyping; Phylop score (< 0, less conserved; 0, neutral; > 0 conserved; a large score indicates high conservation)

#### Rare variants in CD families

We detected four heterozygous overlapping rare sequence variants in four candidate genes (*TFPI*, OMIM 152310; *TLR1*, OMIM, 601194; *COL15A1*; *C1QBP*, OMIM 601269) in affected members of family 1 (Table 3). Family 3 harbored three heterozygous overlapping rare sequence variants in three candidate genes (*DDR1*, OMIM 600408; *VWF*, OMIM 613160; *SLC12A3*, OMIM 600968) (Table 3). Families 2 and 6 harbored one heterozygous overlapping rare variant in one candidate gene each (Family 2: *ADAMTS20*, OMIM 611681; Family 6: *ITGA1*, OMIM 192968), but both variants were not confirmed by Sanger sequencing (S15 Table). No overlapping rare sequence variants were detected in candidate genes in affected members of families 4 and 5 (S2 Table).

**Table 3:** Overlapping rare sequence variants identified in seven of 289 candidate genes in affected members of CD families by WES.

Gene	Change in		dbSNP		Prediction algorithms		Conservation	Family number
	Nucleotide	Protein	ID	MAF (%)	SIFT	PolyPhen2	Phylop (Base level)	
*TFPI*	c.874G>A	p.Val292Met	rs5940	0.008	Deleterious	Damaging	0.5	1
*TLR1*	c.1138C>T	p.Gln380*	NA	0	NA	NA	1.3	1
*COL15A1*	c.2114C>T	p.Pro705Leu	rs41308900	0.007	Deleterious	Damaging	1.9	1
*C1QBP*	c.389C>T	p.Thr130Met	rs56014026	0.007	Tolerated	Damaging	3.4	1
*DDR1*	c.1093A>T	p.Ile365Phe	rs143367160	0.003	Deleterious	Benign	1.6	3
*VWF*	c.2771C>T	p.Arg924Gln	rs33978901	0.007	Tolerated	Benign	1.4	3
*SLC12A3*	c.2883+1 G>T	Splice-donor	rs199974259	0	NA	NA	5.2	3

CD, cuticular drusen; WES, whole exome sequencing; MAF, minor allele frequency; SIFT, sorting intolerant from tolerant; PolyPhen2, polymorphism phenotyping; Phylop score (< 0, less conserved; 0, neutral; > 0 conserved; a large score indicates high conservation); NA, not applicable

**Fig 3:**
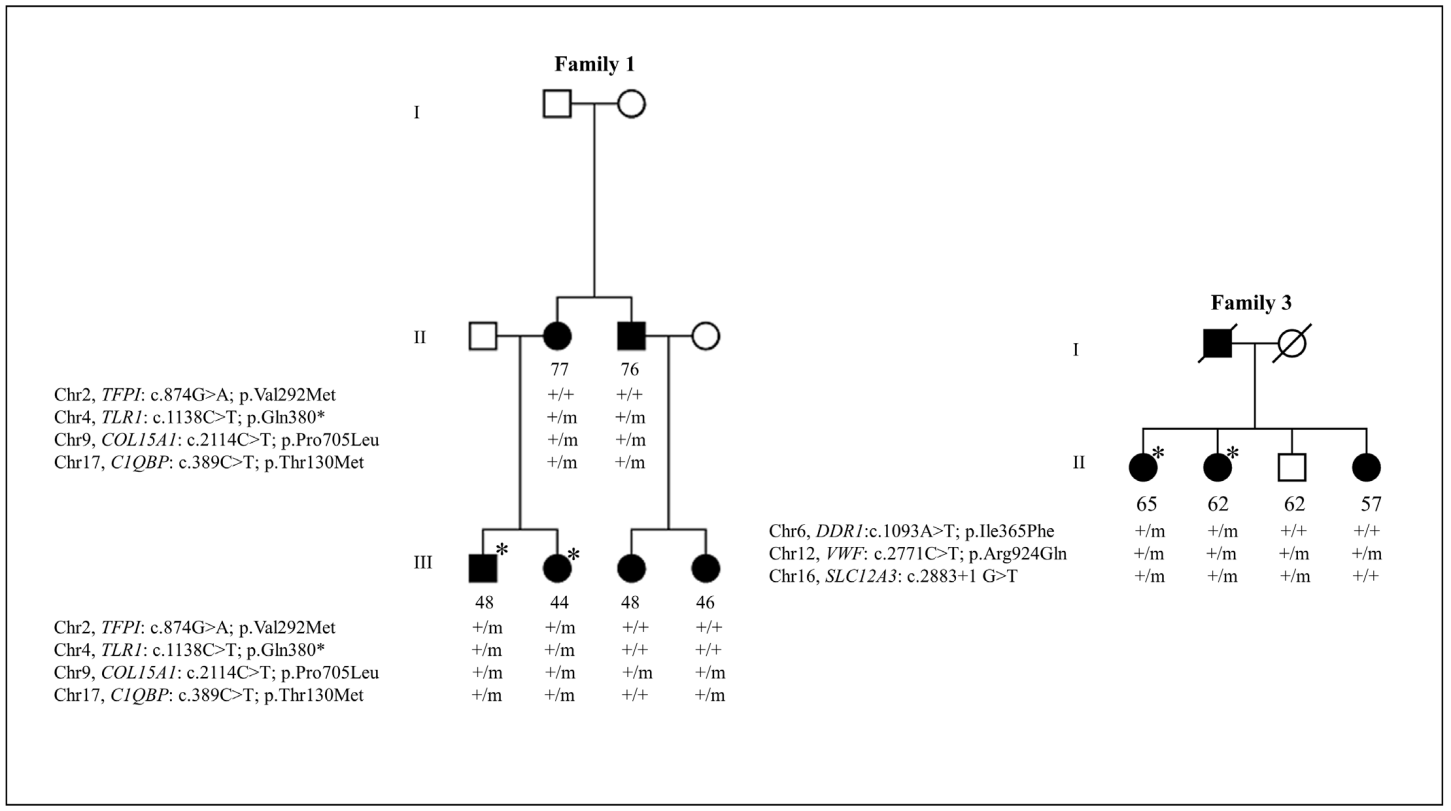
Segregation analysis of rare sequence variants identified in candidate genes in cuticular drusen (CD) families by whole exome sequencing (WES). Circles, females; squares, males; empty symbols, unaffected; black symbols, affected; asterisks, exome sequenced individuals; ‘+’ symbol, wild type allele; ‘m’ symbol, mutant type allele. The age at participation is specified below the symbols.

In family 1, all overlapping rare sequence variants (p.Val292Met/rs5940, *TFPI*; p.Gln380*, *TLR1*; p.Pro705Leu/rs41308900, *COL15A1*; p.Thr130Met/rs56014026, *C1QBP*) in candidate genes were confirmed by Sanger sequencing (Table 3). Two variants, p.Val292Met (*TFPI*) and p.Pro705Leu (*COL15A1*), are predicted to be damaging by both SIFT and PolyPhen2, while a variant p.Thr130Met (*C1QBP*) is predicted to be damaging by one of two algorithms. Evolutionary conservation suggested that variants in the *TFPI*, *TLR1*, *COL15A1*, and *C1QBP* genes are conserved. We next checked for segregation of all confirmed variants with CD in the additional members of family 1 by Sanger sequencing. Variant p.Pro705Leu in the *COL15A1* gene segregated heterozygously with CD in this family, although unaffected family members above age 60 were not available in this family. All other variants were not present in one or two affected members of a family, and thus did not segregate with CD in family 1 (Fig 3).

Family 3 harbored three overlapping rare sequence variants (p.Ile365Phe/rs143367160, *DDR1*; p.Arg924Gln/rs33978901, *VWF*; c.2883+1G>T/rs199974259, *SLC12A3*), which were all confirmed by Sanger sequencing. Variant p.Ile365Phe in the *DDR1* gene is predicted to be damaging by one of two prediction algorithms, while the p.Arg924Gln variant in the *VWF* gene is predicted not to be damaging by both SIFT and PolyPhen2 prediction algorithms. All three variants are evolutionarily conserved. These variants subsequently were analyzed for segregation with CD in two additional family members of family 3. Variant p.Ile365Phe in the *DDR1* gene did not show segregation, as it was not present in an affected member of the family. The splice donor variant, c.2883+1G>T, in the *SLC12A3* gene also did not segregate, as it was found to be present in an unaffected member and was not present in an affected member of the family. The missense variant p.Arg924Gln in the *VWF* gene is present in three affected members and one unaffected member of the family (Fig 3).

The exomes of the six families were also analyzed for variants in all genes of the exome that were shared among all affected individuals of all six families. We filtered the exome data for rare non-synonymous, coding indels, and splice acceptor and donor site variants with a MAF ≤1% in dbSNP and in our in-house exome database. These filtering criteria did not identify any rare sequence variants in a single gene that segregate with CD in the six families.

## Discussion

In the present study, we found rare sequence variants in two genes which previously were shown to harbor rare variants in CD (*CFH* and *FBLN5*) [5,11]. In addition, we found heterozygous rare sequence variants (MAF ≤0.01) in several extracellular matrix (ECM) genes, which include *FBLN1*, *FBLN3*/*EFEMP1*, *FBLN5*, *FBLN6*/*HMCN1*, *FBN2*, and *COL15A1*, in sporadic cases and families with the CD subtype of AMD by WES (Tables 1 and 2). Two rare pathogenic variants were identified in the *COL15A1* gene: one in a sporadic case and another was found to segregate in a family with six affected individuals with CD. In addition, two rare pathogenic variants were identified in the *FGL1* gene in three unrelated CD cases.

Variants in the *CFH* gene are major risk factors for both AMD and CD [10,11,19]. In this study, we identified two missense variants (p.Ala173Gly and p.Gln950His) that were previously identified by Sanger sequencing of the *CFH* gene in the same patients of the CD subtype of AMD phenotype [12].

The fibulins are ECM proteins that are characterized by tandem arrays of epidermal growth factor (EGF)—like domains, and are widely expressed in basement membranes. A previous study suggests that a single mutation (p.Arg345Trp) in the last EGF domain of *FBLN3*/*EFEMP1* gene causes Doyne honeycomb retinal dystrophy (DHRD; OMIM 126600) [21]. We identified an evolutionarily conserved rare sequence variant (p.Asp49Ala) in the first EGF domain of *FBLN3*/*EFEMP1* gene in a sporadic CD patient. This suggests that variants in the *FBLN3*/*EFEMP1* gene may represent risk factors for the CD phenotype. We also identified a missense variant (p.Val126Met) in the *FBLN5* gene, which was previously reported in the Dutch population (AMD cases 1/291; controls 5/91), suggesting that this variant is not rare in the Dutch population [22]. Therefore, the p.Val126Met variant in the *FBLN5* gene may not be a causal variant for CD. However, missense variants in the *FBLN5* gene have previously been associated with AMD (*P* < 0.01) and the retinal images of patients showed a peculiar CD phenotype, which suggests that other variants in the *FBLN5* gene are a risk factor for both the AMD and CD phenotypes [5,22].

The ECM is an acellular component that provides physical and biochemical support for surrounding cells in all tissues and organs, and constantly undergoes remodeling processes which are indispensible for tissue architecture [23]. A pathological symptom of AMD phenotypes is the formation of drusen between the retinal pigment epithelium (RPE) and Bruch’s membrane (BrM), which is a multilayered ECM structure. The BrM is composed of central elastin fibers sandwiched between layers of collagen [24,25]. The BrM acts as a blood-retinal barrier that regulates the diffusion of nutrients and oxygen from the choroid through the BrM to the RPE, while the metabolic waste diffuses in an opposite direction to the choroid [26]. Several lines of evidence suggest that ageing processes (thickening, calcification, degeneration of collagens and elastic fibers) and alterations in the structural components in the BrM result in loss of normal function of the BrM [27], which may result in accumulation of drusen deposits in AMD patients. Emerging evidence suggests that alterations in specific genes encoding ECM proteins (TIMP3, CTRP5, FBN2, and FBLN 1–6) are implicated in macular degeneration [5,21,28–30]. Recent genome-wide association studies have also identified risk variants in several ECM genes (*COL8A1*, *COL10A1*, *ADAMTS9*, *DDR1*, *TGFBR1*, *HTRA1*, and *TIMP3*) [20].

The present study identified two evolutionarily conserved rare sequence variants (p.Pro705Leu and p.Phe851Leu) in *COL15A1* in 6 affected members of a family and in a sporadic CD patient, respectively. The p.Pro705Leu variant is predicted to be deleterious by both prediction algorithms, while the p.Phe851Leu variant is predicted to be deleterious by one of two prediction algorithms. These prediction algorithms provide a quick functional annotation of variants, but experimental validation is required to properly access the functional consequences of these variants. The *COL15A1* gene encodes collagen, type XV, alpha 1, which is widely expressed in basement membranes [31], and in choroidal endothelial cells [32]. Since the choroid plays a vital role in maintenance of BrM, variants in the *COL15A1* gene might lead to altered properties of the choroid and/or of BrM, resulting in the formation of drusen in patients with CD.

In this study we also identified two highly conserved rare sequence variants in the *FGL1* gene in three of 12 sporadic CD cases (Table 2). Both variants are predicted to be deleterious to the normal function of the protein by prediction algorithms. Fibrinogen and fibrinogen-like protein 1 (FGL1) belong to the fibrinogen superfamily. Fibrinogen is a precursor of fibrin clot formation of the coagulation cascade [33,34]. A proteomic study demonstrated that FGL1 is bound to the fibrin matrix during clot formation, suggesting that FGL1 is involved in the coagulation cascade [35,36]. Interestingly, the molecular composition of drusen consist of fibrinogen as one of several constituents [37]. This suggests that *FGL1* variants may be causative in CD, although it is unclear on how the coagulation cascade and variants in *FGL1* gene may be implicated in AMD phenotypes.

We found no segregating rare variants among affected members of four of the six CD families in the 289 candidate genes. In all protein-coding regions of the genome, we also found no evidence of rare sequence variants in a single gene that segregate with CD in all families, providing evidence for genetic heterogeneity in the pathogenesis of CD. Alternatively, the disease risk in some individuals of these families may be attributable to a combination of common genetic and environmental factors, and as a consequence rare variants may not completely segregate in these families. In a recent study we identified a clustering of known risk factors in affected members of families with AMD, suggesting that such families may not be explained by rare genetic variants. However, some families cannot be explained by known risk factors, and are more likely to carry rare, highly penetrant variants [38]. Some affected family members in the CD families examined in this study might thus have CD due to the presence of known risk factors, and not merely by rare sequence variants. Therefore, WES based segregation analyses may not always be the best strategy to solve AMD or CD families. However, other studies have successfully identified rare sequence variants in AMD families by WES, e.g. in the *CFH* and *FBN2* genes [28,39].

In summary, WES in sporadic cases and families with the CD subtype of AMD identified rare variants in known CD genes and several genes encoding ECM components. Rare pathogenic variants were recurrently identified in the *COL15A1* and *FGL1* genes. These findings suggest that alterations in the ECM and in the coagulation pathway may play a role in the pathogenesis of CD. These candidate genes require further analyses in larger cohorts to confirm their involvement in the CD subtype of AMD. No evidence was found of rare sequence variants in a single gene that segregate with CD in the six families, suggesting that the disease is genetically heterogeneous.

## Supporting Information

S1 Table List of 289 candidate genes. (DOCX)

S2 Table Whole exome sequencing filtering. (DOCX)

S3 Table Sporadic case 1AB, Fig 2. (DOCX)

S4 Table Sporadic case 2AB, Fig 2. (DOCX)

S5 Table Sporadic case 3AB, Fig 2. (DOCX)

S6 Table Sporadic case 4AB, Fig 2. (DOCX)

S7 Table Sporadic case 5AB, Fig 2. (DOCX)

S8 Table Sporadic case 6AB, Fig 2. (DOCX)

S9 Table Sporadic case 7AB, Fig 2. (DOCX)

S10 Table Sporadic case 8AB, Fig 2. (DOCX)

S11 Table Sporadic case 9AB, Fig 2. (DOCX)

S12 Table Sporadic case 10AB, Fig 2. (DOCX)

S13 Table Sporadic case 11AB, Fig 2. (DOCX)

S14 Table Sporadic case 12AB, Fig 2. (DOCX)

S15 Table Unconfirmed rare sequence variants. (DOCX)
