# Supplementary material for: Whole Exome Sequencing in Patients with the Cuticular Drusen Subtype of Age-Related Macular Degeneration
Source: PLoS One. 2016 Mar 23;11(3):e0152047. doi: 10.1371/journal.pone.0152047 (PMC4805164; doi:10.1371/journal.pone.0152047)
Supplement: S1 Table — (DOCX) [file pone.0152047.s001.docx]

**S1 Table**. **List of 289 candidate genes**

List of 289 candidate genes selected from known AMD loci, genes known to be involved in monogenic macular degeneration, genes from the complement system, the coagulation system, innate immunity, endothelial cell components, and glomerular basement membrane components (Westra *et al*, in preparation).

| **Gene** | **ID** | **OMIM** | **Gene** | **ID** | **OMIM** | **Gene** | **ID** | **OMIM** | **Gene** | **ID** | **OMIM** | **Gene** | **ID** | **OMIM** |
| --- | --- | --- | --- | --- | --- | --- | --- | --- | --- | --- | --- | --- | --- | --- |
| *A2M* | 2 | 103950 | *C1R* | 715 | 613785 | *CFD* | 1675 | 134350 | *F2* | 2147 | 176930 | *GPC6* | 10082 | 604404 |
| *ABCA4* | 24 | 601691 | *C1RL* | 51279 | 608974 | *CFH* | 3075 | 134370 | *F2R* | 2149 | 187930 | *GPR108* | 56927 | NA |
| *ACAN* | 176 | 155760 | *C1S* | 716 | 120580 | *CFHR1* | 3078 | 134371 | *F3* | 2152 | 134390 | *GTF2H4* | 2968 | 601760 |
| *ACE* | 1636 | 106180 | *C2* | 717 | 613927 | *CFHR2* | 3080 | 600889 | *F5* | 2153 | 612309 | *GUCA1B* | 2979 | 602275 |
| *ADAMTS13* | 11093 | 604134 | *C3* | 718 | 120700 | *CFHR3* | 10878 | 605336 | *F7* | 2155 | 613878 | *HERPUD1* | 9709 | 608070 |
| *ADAMTS17* | 170691 | 607511 | *C3AR1* | 719 | 605246 | *CFHR4* | 10877 | 605337 | *F8* | 2157 | 300841 | *HMCN1* | 83872 | 608548 |
| *ADAMTS19* | 171019 | 607513 | *C4A* | 720 | 120810 | *CFHR5* | 81494 | 608593 | *F9* | 2158 | 300746 | *HSPG2* | 3339 | 142461 |
| *ADAMTS2* | 9509 | 604539 | *C4B* | 721 | 120820 | *CFI* | 3426 | 217030 | *FBLN5* | 10516 | 604580 | *HSPH1* | 10808 | 610703 |
| *ADAMTS20* | 80070 | 611681 | *C4BPA* | 722 | 120830 | *CFP* | 5199 | 300383 | *FBN2* | 2201 | 612570 | *HTRA1* | 5654 | 602194 |
| *ADAMTS9* | 56999 | 605421 | *C4BPB* | 725 | 120831 | *CHMP7* | 91782 | 611130 | *FCN1* | 2219 | 601252 | *ICAM1* | 3383 | 147840 |
| *AGT* | 183 | 106150 | *C5* | 727 | 120900 | *CLPTM1* | 1209 | 604783 | *FCN2* | 2220 | 601624 | *ICAM2* | 3384 | 146630 |
| *AGTR1* | 185 | 106165 | *C5AR1* | 728 | 113995 | *CLU* | 1191 | 185430 | *FCN3* | 8547 | 604973 | *ICAM3* | 3385 | 146631 |
| *ALG2* | 85365 | 607905 | *C6* | 729 | 217050 | *COL10A1* | 1300 | 120110 | *FGA* | 2243 | 134820 | *ICAM4* | 3386 | 614088 |
| *APOB* | 338 | 107730 | *C6orf223* | 221416 | NA | *COL15A1* | 1306 | 120325 | *FGB* | 2244 | 134830 | *IER3* | 8870 | 602996 |
| *APOC1* | 341 | 107710 | *C7* | 730 | 217070 | *COL8A1* | 1295 | 120251 | *FGD5* | 152273 | 614788 | *IFNG* | 3458 | 147570 |
| *APOC1P1* | 342 | NA | *C8A* | 731 | 120950 | *CPB2* | 1361 | 603101 | *FGD6* | 55785 | 613520 | *IL10* | 3586 | 124092 |
| *APOC2* | 344 | 608083 | *C8B* | 732 | 120960 | *CR1* | 1378 | 120620 | *FGG* | 2266 | 134850 | *IL12A* | 3592 | 161560 |
| *APOC4* | 346 | 600745 | *C8G* | 733 | 120930 | *CR1L* | 1379 | 605886 | *FGL1* | 2267 | 605776 | *IL12RB1* | 3594 | 601604 |
| *APOE* | 348 | 107741 | *C9* | 735 | 120940 | *CR2* | 1380 | 120650 | *FGL2* | 10875 | 605351 | *IL1A* | 3552 | 147760 |
| *ARMS2* | 387715 | 611313 | *CASP6* | 839 | 601532 | *CSMD1* | 64478 | 608397 | *FGR* | 2268 | 164940 | *IL2* | 3558 | 147680 |
| *B3GALTL* | 145173 | 610308 | *CCDC109B* | 55013 | NA | *CX3CR1* | 1524 | 601470 | *FIGF* | 2277 | 300091 | *IL6* | 3569 | 147620 |
| *BAIAP2L2* | 80115 | NA | *CD14* | 929 | 158120 | *CYP21A2* | 1589 | 613815 | *FILIP1L* | 11259 | 612993 | *IMPG1* | 3617 | 602870 |
| *BCAM* | 4059 | 612773 | *CD19* | 930 | 107265 | *DAPL1* | 92196 | NA | *FLOT1* | 10211 | 606998 | *ITGA1* | 3672 | 192968 |
| *BDKRB1* | 623 | 600337 | *CD36* | 948 | 173510 | *DDR1* | 780 | 600408 | *FLT1* | 2321 | 165070 | *ITGA10* | 8515 | 604042 |
| *BDKRB2* | 624 | 113503 | *CD40* | 958 | 109535 | *DGKE* | 8526 | 601440 | *FLT4* | 2324 | 136352 | *ITGA11* | 22801 | 604789 |
| *BEST1* | 7439 | 607854 | *CD44* | 960 | 107269 | *DMBT1* | 1755 | 601969 | *FN1* | 2335 | 135600 | *ITGA2* | 3673 | 192974 |
| *C1QA* | 712 | 120550 | *CD46* | 4179 | 120920 | *EFEMP1* | 2202 | 601548 | *FRK* | 2444 | 606573 | *ITGA2B* | 3674 | 607759 |
| *C1QB* | 713 | 120570 | *CD55* | 1604 | 125240 | *EHMT2* | 10919 | 604599 | *FSCN2* | 25794 | 607643 | *ITGA3* | 3675 | 605025 |
| *C1QBP* | 708 | 601269 | *CD59* | 966 | 107271 | *ELOVL4* | 6785 | 605512 | *GP1BA* | 2811 | 606672 | *ITGA4* | 3676 | 192975 |
| *C1QC* | 714 | 120575 | *CD74* | 972 | 142790 | *ERCC6* | 2074 | 609413 | *GP6* | 51206 | 605546 | *ITGA5* | 3678 | 135620 |
| *C1QL1* | 10882 | 611586 | *CD79A* | 973 | 112205 | *F10* | 2159 | 613872 | *GPC1* | 2817 | 600395 | *ITGA6* | 3655 | 147556 |
| *C1QL2* | 165257 | 614330 | *CD79B* | 974 | 147245 | *F11* | 2160 | 264900 | *GPC2* | 221914 | NA | *ITGA7* | 3679 | 600536 |
| *C1QL3* | 389941 | 615227 | *CD93* | 22918 | 120577 | *F12* | 2161 | 610619 | *GPC3* | 2719 | 300037 | *ITGA8* | 8516 | 604063 |
| *C1QL4* | 338761 | 615229 | *CETP* | 1071 | 118470 | *F13A1* | 2162 | 134570 | *GPC4* | 2239 | 300168 | *ITGA9* | 3680 | 603963 |
| *C1QTNF5* | 114902 | 608752 | *CFB* | 629 | 138470 | *F13B* | 2165 | 134580 | *GPC5* | 2262 | 602446 | *ITGAD* | 3681 | 602453 |

**S1 Table continued**.

| **Gene** | **ID** | **OMIM** | **Gene** | **ID** | **OMIM** | **Gene** | **ID** | **OMIM** | **Gene** | **ID** | **OMIM** |
| --- | --- | --- | --- | --- | --- | --- | --- | --- | --- | --- | --- |
| *ITGAE* | 3682 | 604682 | *PDGFA* | 5154 | 173430 | *SERPINE1* | 5054 | 173360 | *TUBB* | 203068 | 191130 |
| *ITGAL* | 3683 | 153370 | *PDGFB* | 5155 | 190040 | *SERPINF2* | 5345 | 613168 | *VARS2* | 57176 | 612802 |
| *ITGAM* | 3684 | 120980 | *PDGFRA* | 5156 | 173490 | *SERPING1* | 710 | 606860 | *VAV1* | 7409 | 164875 |
| *ITGAV* | 3685 | 193210 | *PDGFRB* | 5159 | 173410 | *SH2D3A* | 10045 | 604721 | *VEGFA* | 7422 | 192240 |
| *ITGAW* | 3686 | NA | *PGF* | 5228 | 601121 | *SKIV2L* | 6499 | 600478 | *VEGFB* | 7423 | 601398 |
| *ITGAX* | 3687 | 151510 | *PICK1* | 9463 | 605926 | *SLC12A3* | 6559 | 600968 | *VEGFC* | 7424 | 601528 |
| *ITGB1* | 3688 | 135630 | *PLA2G12A* | 81579 | 611652 | *SLC16A8* | 23539 | 610409 | *VTN* | 7448 | 193190 |
| *ITGB2* | 3689 | 600065 | *PLA2G6* | 8398 | 603604 | *SLC44A4* | 80736 | 606107 | *VWF* | 7450 | 613160 |
| *ITGB3* | 3690 | 173470 | *PLAT* | 5327 | 173370 | *SOX10* | 6663 | 602229 | *ZBTB12* | 221527 | NA |
| *ITGB4* | 3691 | 147557 | *PLAU* | 5328 | 191840 | *STK19* | 8859 | 604977 |  |  |  |
| *ITGB5* | 3693 | 147561 | *PLAUR* | 5329 | 173391 | *SUSD4* | 55061 | 615827 |  |  |  |
| *ITGB6* | 3694 | 147558 | *PLEKHA1* | 59338 | 607772 | *SYN3* | 8224 | 602705 |  |  |  |
| *ITGB7* | 3695 | 147559 | *PLG* | 5340 | 173350 | *TFPI* | 7035 | 152310 |  |  |  |
| *ITGB8* | 3696 | 604160 | *PROC* | 5624 | 612283 | *TGFB1* | 7040 | 190180 |  |  |  |
| *KDR* | 3791 | 191306 | *PROCR* | 10544 | 600646 | *TGFBR1* | 7046 | 190181 |  |  |  |
| *KLKB1* | 3818 | 229000 | *PROM1* | 8842 | 604365 | *THBD* | 7056 | 188040 |  |  |  |
| *KNG1* | 3827 | 612358 | *PROS1* | 5627 | 176880 | *TIMP3* | 7078 | 188826 |  |  |  |
| *LIPC* | 3990 | 151670 | *PROZ* | 8858 | 176895 | *TLR1* | 7096 | 601194 |  |  |  |
| *LOC285858* | 285858 | NA | *PRPH2* | 5961 | 179605 | *TLR2* | 7097 | 603028 |  |  |  |
| *LOC389641* | 389641 | NA | *PVRL2* | 5819 | 600798 | *TLR3* | 7098 | 603029 |  |  |  |
| *LOXL2* | 4017 | 606663 | *R3HCC1* | 203069 | NA | *TLR4* | 7099 | 603030 |  |  |  |
| *LY96* | 23643 | 605243 | *RAD51B* | 5890 | 602948 | *TLR5* | 7100 | 603031 |  |  |  |
| *MASP1* | 5648 | 600521 | *RAX2* | 84839 | 610362 | *TLR6* | 10333 | 605403 |  |  |  |
| *MASP2* | 10747 | 605102 | *RELB* | 5971 | 604758 | *TLR7* | 51284 | 300365 |  |  |  |
| *MBL2* | 4153 | 154545 | *RP1L1* | 94137 | 608581 | *TLR8* | 51311 | 300366 |  |  |  |
| *MDC1* | 9656 | 607593 | *RPGR* | 6103 | 312610 | *TNF* | 7124 | 191160 |  |  |  |
| *MIF* | 4282 | 153620 | *SDC1* | 6382 | 186355 | *TNFRSF10A* | 8797 | 603611 |  |  |  |
| *MIR1236* | 100302242 | NA | *SDC2* | 6383 | 142460 | *TNFRSF10D* | 8793 | 603614 |  |  |  |
| *MIR3941* | 100500866 | NA | *SEC24B* | 10427 | 607184 | *TNFSF14* | 8740 | 604520 |  |  |  |
| *MIR548A2* | 693126 | NA | *SEC61B* | 10952 | 609214 | *TNXA* | 7146 | NA |  |  |  |
| *MIR548G* | 100313938 | NA | *SERPINA1* | 5265 | 107400 | *TNXB* | 7148 | 600985 |  |  |  |
| *MMACHC* | 25974 | 609831 | *SERPINA5* | 5104 | 601841 | *TOMM40* | 10452 | 608061 |  |  |  |
| *NEU1* | 4758 | 608272 | *SERPINB2* | 5055 | 173390 | *TPI1P3* | 728402 | NA |  |  |  |
| *NLRC5* | 84166 | 613537 | *SERPINC1* | 462 | 107300 | *TRIP10* | 9322 | 604504 |  |  |  |
| *NT5DC1* | 221294 | NA | *SERPIND1* | 3053 | 142360 | *TSPYL4* | 23270 | NA |  |  |  |
