# Supplementary material for: Whole Exome Sequencing in Patients with the Cuticular Drusen Subtype of Age-Related Macular Degeneration
Source: PLoS One. 2016 Mar 23;11(3):e0152047. doi: 10.1371/journal.pone.0152047 (PMC4805164; doi:10.1371/journal.pone.0152047)
Supplement: S2 Table — (DOCX) [file pone.0152047.s002.docx]

**S2 Table. Whole exome sequencing filtering**

Whole exome sequencing filtering steps for 289 candidate genes in 14 affected members of six CD families and 12 sporadic CD cases

| **Samples** | **# Variants in** | **Variant reads** | | **Variants in** | **NS/I/SS** | **db SNP** |
| --- | --- | --- | --- | --- | --- | --- |
|  | **candidate genes** | **≥ 10** | **≥ 20%** | **exons** | **variants** | **MAF (≤ 0.01)** |
| **Families (Figure 1)** | **# Overlapping variants** |  |  |  |  |  |
| Family 1 – two FM | 615 | 496 | 491 | 232 | 108 | 4 |
| Family 2 – two FM | 439 | 326 | 323 | 160 | 69 | 1 |
| Family 3 – two FM | 549 | 414 | 413 | 196 | 90 | 3 |
| Family 4 – four FM | 345 | 307 | 305 | 149 | 71 | 0 |
| Family 5 – two FM | 517 | 405 | 404 | 188 | 86 | 0 |
| Family 6 – two FM | 586 | 447 | 445 | 230 | 103 | 1 |
| **Average** | 508 ± 100 | 399 ± 71 | 397 ± 71 | 192 ± 34 | 88 ± 16 | 2 ± 2 |
| **Sporadic (Figure 2)** |  |  |  |  |  |  |
| 1AB | 675 | 421 | 419 | 209 | 105 | 5 |
| 2AB | 767 | 533 | 526 | 277 | 132 | 6 |
| 3AB | 864 | 661 | 655 | 343 | 163 | 10 |
| 4AB | 839 | 642 | 638 | 298 | 151 | 12 |
| 5AB | 799 | 551 | 546 | 284 | 130 | 6 |
| 6AB | 770 | 585 | 581 | 253 | 122 | 8 |
| 7AB | 716 | 467 | 461 | 231 | 108 | 8 |
| 8AB | 722 | 517 | 514 | 266 | 136 | 8 |
| 9AB | 754 | 515 | 508 | 272 | 122 | 9 |
| 10AB | 836 | 637 | 629 | 327 | 154 | 9 |
| 11AB | 760 | 555 | 553 | 275 | 132 | 10 |
| 12AB | 856 | 618 | 611 | 307 | 148 | 7 |
| **Average** | 780 ± 60 | 558 ± 73 | 553 ± 72 | 278 ± 37 | 133 ± 18 | 8 ± 2 |

NS, nonsynonymous variants; I, coding indels; SS, splice acceptor and donor site variants; FM, family members; Families 4 & 5, no overlapping variants detected; Variant reads,

variants were selected when they were present on 10 or more (≥ 10) variant reads, and when they were present in 20 percent or more (≥ 20%) of the variant reads.
