# Supplementary material for: Whole Exome Sequencing in Patients with the Cuticular Drusen Subtype of Age-Related Macular Degeneration
Source: PLoS One. 2016 Mar 23;11(3):e0152047. doi: 10.1371/journal.pone.0152047 (PMC4805164; doi:10.1371/journal.pone.0152047)
Supplement: S3 Table — (DOCX) [file pone.0152047.s003.docx]

**S3 Table. Sporadic case 1AB, Fig 2**

| **Chromosome** | | **Gene** | **Change in** | | **SNP id** | **MAF** | **Conservation** |
| --- | --- | --- | --- | --- | --- | --- | --- |
| **#** | **Position** |  | **Nucleotide** | **Amino acid** |  |  | **Phylop (Base level)** |
| 3 | 37560750 | *ITGA9* | 1142-1 G>A | NA | NA | 0 | 6.26 |
| 4 | 177605082 | *VEGFC* | 1258TCA> | S420 | rs5864401 | 0.003 | 2 |
| 12 | 9246177 | *A2M* | 2126-6_2126-2 | NA | rs3832852 | 0 | 0.88 |
| 17 | 48152859 | *ITGA3* | 1506T>G | S502R | NA | 0 | 0.78 |
| 19 | 18192977 | *IL12RB1* | 222G>C | S74R | rs11575925 | 0.0004 | -0.08 |

MAF, Minor Allele Frequency; Phylop score (< 0, less conserved; 0, neutral; > 0 conserved; a large score indicates high conservation)
