# Supplementary material for: Whole Exome Sequencing in Patients with the Cuticular Drusen Subtype of Age-Related Macular Degeneration
Source: PLoS One. 2016 Mar 23;11(3):e0152047. doi: 10.1371/journal.pone.0152047 (PMC4805164; doi:10.1371/journal.pone.0152047)
Supplement: S4 Table — (DOCX) [file pone.0152047.s004.docx]

**S4 Table. Sporadic case 2AB, Fig 2**

| **Chromosome** | | **Gene** | **Change in** | | **SNP id** | **MAF** | **Conservation** |
| --- | --- | --- | --- | --- | --- | --- | --- |
| **#** | **Position** |  | **Nucleotide** | **Amino acid** |  |  | **Phylop (Base level)** |
| 3 | 186959295 | *MASP1* | 1277C>T | G426E | rs28945068 | 0.008 | 1.74 |
| 4 | 177605082 | *VEGFC* | 1258TCA> | S420 | rs5864401 | 0.003 | 2 |
| 5 | 52225518 | *ITGA1* | 2758T>G | L920V | NA | 0 | 0.12 |
| 6 | 6251120 | *F13A1* | 614T>A | Y205F | rs3024477 | 0.008 | 1.88 |
| 8 | 23148940 | *R3HCC1* | 358G>T | V120L | NA | 0 | 0.02 |
| 9 | 101804366 | *COL15A1* | 2551T>C | F851L | rs35901514 | 0.003 | 2.87 |

MAF, Minor Allele Frequency; Phylop score (< 0, less conserved; 0, neutral; > 0 conserved; a large score indicates high conservation)
