# Supplementary material for: Whole Exome Sequencing in Patients with the Cuticular Drusen Subtype of Age-Related Macular Degeneration
Source: PLoS One. 2016 Mar 23;11(3):e0152047. doi: 10.1371/journal.pone.0152047 (PMC4805164; doi:10.1371/journal.pone.0152047)
Supplement: S5 Table — (DOCX) [file pone.0152047.s005.docx]

**S5 Table. Sporadic case 3AB, Fig 2**

| **Chromosome** | | **Gene** | **Change in** | | **SNP id** | **MAF** | **Conservation** |
| --- | --- | --- | --- | --- | --- | --- | --- |
| **#** | **Position** |  | **Nucleotide** | **Amino acid** |  |  | **Phylop (Base level)** |
| 1 | 196646696 | *CFH* | 518C>G | A173G | NA | 0 | 0.31 |
| 3 | 99649861 | *FILIP1L* | 4G>T | R2S | NA | 0 | 2.86 |
| 4 | 177605082 | *VEGFC* | 1258TCA> | S420 | rs5864401 | 0.003 | 2 |
| 6 | 30866675 | *DDR1* | 2480C>T | T827M | rs150642742 | 0.0002 | 5.24 |
| 6 | 80634724 | *ELOVL4* | 314C>G | G105A | NA | 0 | 2.41 |
| 7 | 22771038 | *IL6* | 485A>T | D162V | rs2069860 | 0.003 | 0.12 |
| 12 | 56079053 | *ITGA7* | 2924G>A | A975V | rs139136931 | 0.0008 | 2.31 |
| 12 | 7242224 | *C1R* | 530 C>T | R177H | NA | 0 | -0.1 |
| 16 | 31336059 | *ITGAM* | 2248C>T | R750W | NA | 0 | 1.85 |
| 19 | 55525763 | *GP6* | 1550A>C | M517R | rs200566792 | 0.0008 | -0.89 |

MAF, Minor Allele Frequency; Phylop score (< 0, less conserved; 0, neutral; > 0 conserved; a large score indicates high conservation)
