# Supplementary material for: Whole Exome Sequencing in Patients with the Cuticular Drusen Subtype of Age-Related Macular Degeneration
Source: PLoS One. 2016 Mar 23;11(3):e0152047. doi: 10.1371/journal.pone.0152047 (PMC4805164; doi:10.1371/journal.pone.0152047)
Supplement: S6 Table — (DOCX) [file pone.0152047.s006.docx]

**S6 Table. Sporadic case 4AB, Fig 2**

| **Chromosome** | | **Gene** | **Change in** | | **SNP id** | **MAF** | **Conservation** |
| --- | --- | --- | --- | --- | --- | --- | --- |
| **#** | **Position** |  | **Nucleotide** | **Amino acid** |  |  | **Phylop (Base level)** |
| 1 | 145530939 | *ITGA10* | 671C>T | T224M | rs146565671 | 0.002 | 3.38 |
| 3 | 186959295 | *MASP1* | 1277C>T | G426E | rs28945068 | 0.008 | 1.74 |
| 3 | 186974474 | *MASP1* | 722G>A | P241L | NA | 0 | 2.91 |
| 4 | 55127448 | *PDGFRA* | 236G>A | G79D | rs36035373 | 0.004 | 4.95 |
| 4 | 177605082 | *VEGFC* | 1258TCA> | S420 | rs5864401 | 0.003 | 2 |
| 6 | 116446506 | *COL10A1* | 150A>T | S50R | rs142411445 | 0.001 | 0.12 |
| 6 | 30680124 | *MDC1* | 1595C>T | G532E | rs139338660 | 0.0004 | 0.37 |
| 8 | 17726069 | *FGL1* | 767C>A | W256L | rs2653414 | 0.009 | 5.17 |
| 12 | 53586255 | *ITGB7* | 2014G>A | H672Y | rs11539433 | 0.004 | 0.31 |
| 12 | 95603246 | *FGD6* | 1814G>A | S605L | rs200713610 | 0.0006 | 4.52 |
| 12 | 54793659 | *ITGA5* | 2716G>C | P906A | NA | 0 | 0.3 |
| 13 | 31729776 | *HSPH1* | 181T>G | N61H | rs41292149 | 0.002 | 3.15 |

MAF, Minor Allele Frequency; Phylop score (< 0, less conserved; 0, neutral; > 0 conserved; a large score indicates high conservation)
