# Supplementary material for: Whole Exome Sequencing in Patients with the Cuticular Drusen Subtype of Age-Related Macular Degeneration
Source: PLoS One. 2016 Mar 23;11(3):e0152047. doi: 10.1371/journal.pone.0152047 (PMC4805164; doi:10.1371/journal.pone.0152047)
Supplement: S7 Table — (DOCX) [file pone.0152047.s007.docx]

**S7 Table. Sporadic case 5AB, Fig 2**

| **Chromosome** | | **Gene** | **Change in** | | **SNP id** | **MAF** | **Conservation** |
| --- | --- | --- | --- | --- | --- | --- | --- |
| **#** | **Position** |  | **Nucleotide** | **Amino acid** |  |  | **Phylop (Base level)** |
| 1 | 207300070 | *C4BPA* | 719G>A | R240H | rs45574833 | 0.003 | -0.77 |
| 3 | 39307832 | *CX3CR1* | 265T>C | T89A | rs199811198 | 0.0008 | 0.22 |
| 4 | 177605082 | *VEGFC* | 1258TCA> | S420 | rs5864401 | 0.003 | 2 |
| 10 | 124396776 | *DMBT1* | 6473C>T | P2158L | NA | 0 | -0.27 |
| 16 | 56921840 | *SLC12A3* | 2179G>A | A727T | rs36049418 | 0.006 | -0.37 |
| 17 | 54939158 | *DGKE* | 1291C>G | L431V | NA | 0 | 0.99 |

MAF, Minor Allele Frequency; Phylop score (< 0, less conserved; 0, neutral; > 0 conserved; a large score indicates high conservation)
