# Supplementary material for: Whole Exome Sequencing in Patients with the Cuticular Drusen Subtype of Age-Related Macular Degeneration
Source: PLoS One. 2016 Mar 23;11(3):e0152047. doi: 10.1371/journal.pone.0152047 (PMC4805164; doi:10.1371/journal.pone.0152047)
Supplement: S8 Table — (DOCX) [file pone.0152047.s008.docx]

**S8 Table. Sporadic case 6AB, Fig 2**

| **Chromosome** | | **Gene** | **Change in** | | **SNP id** | **MAF** | **Conservation** |
| --- | --- | --- | --- | --- | --- | --- | --- |
| **#** | **Position** |  | **Nucleotide** | **Amino acid** |  |  | **Phylop (Base level)** |
| 2 | 173355800 | *ITGA6* | 2728G>A | D910N | rs61737182 | 0.002 | 2.1 |
| 4 | 187194331 | *F11* | 325G>A | A109T | NA | 0 | 2.51 |
| 4 | 177605082 | *VEGFC* | 1258TCA> | S420 | rs5864401 | 0.003 | 2 |
| 5 | 52225518 | *ITGA1* | 2758T>G | L920V | NA | 0 | 0.12 |
| 8 | 17726069 | *FGL1* | 767C>A | W256L | rs2653414 | 0.009 | 5.17 |
| 9 | 136280025 | *REXO4* | 332G>A | S111L | rs141547732 | 0.002 | 1.08 |
| 10 | 124175436 | *PLEKHA1* | 530G>A | S177N | rs142473166 | 0.0006 | 4.07 |
| 12 | 43821128 | *ADAMTS20* | 4090A>T | Y1364N | rs138035327 | 0.0004 | 1.57 |

MAF, Minor Allele Frequency; Phylop score (< 0, less conserved; 0, neutral; > 0 conserved; a large score indicates high conservation)
