# Supplementary material for: Whole Exome Sequencing in Patients with the Cuticular Drusen Subtype of Age-Related Macular Degeneration
Source: PLoS One. 2016 Mar 23;11(3):e0152047. doi: 10.1371/journal.pone.0152047 (PMC4805164; doi:10.1371/journal.pone.0152047)
Supplement: S9 Table — (DOCX) [file pone.0152047.s009.docx]

**S9 Table. Sporadic case 7AB, Fig 2**

| **Chromosome** | | **Gene** | **Change in** | | **SNP id** | **MAF** | **Conservation** |
| --- | --- | --- | --- | --- | --- | --- | --- |
| **#** | **Position** |  | **Nucleotide** | **Amino acid** |  |  | **Phylop (Base level)** |
| 2 | 21230743 | *APOB* | 8997G>T | H2999Q | NA | 0 | -0.03 |
| 4 | 38798294 | *TLR1* | 2159T>G | H720P | rs113706342 | 0.004 | 1.09 |
| 4 | 177605082 | *VEGFC* | 1258TCA> | S420 | rs5864401 | 0.003 | 2 |
| 5 | 52225518 | *ITGA1* | 2758T>G | L920V | NA | 0 | 0.12 |
| 5 | 127668685 | *FBN2* | 4141G>T | H1381N | rs78727187 | 0 | 5.91 |
| 6 | 30893728 | *VARS2* | 3123C>G | D1041E | NA | 0 | -0.06 |
| 6 | 30680512 | *MDC1* | 1207C>T | D403N | NA | 0 | 1.77 |
| 16 | 31434770 | *ITGAD* | 2957C>T | A986V | rs150163548 | 0.0004 | 0.09 |

MAF, Minor Allele Frequency; Phylop score (< 0, less conserved; 0, neutral; > 0 conserved; a large score indicates high conservation)
