# Supplementary material for: Whole Exome Sequencing in Patients with the Cuticular Drusen Subtype of Age-Related Macular Degeneration
Source: PLoS One. 2016 Mar 23;11(3):e0152047. doi: 10.1371/journal.pone.0152047 (PMC4805164; doi:10.1371/journal.pone.0152047)
Supplement: S10 Table — (DOCX) [file pone.0152047.s010.docx]

**S10 Table. Sporadic case 8AB, Fig 2**

| **Chromosome** | | **Gene** | **Change in** | | **SNP id** | **MAF** | **Conservation** |
| --- | --- | --- | --- | --- | --- | --- | --- |
| **#** | **Position** |  | **Nucleotide** | **Amino acid** |  |  | **Phylop (Base level)** |
| 1 | 145530980 | *ITGA10* | 712C>T | R238W | rs140344269 | 0.0006 | 0.007 |
| 3 | 99514829 | *COL8A1* | 2084G>A | G695D | rs150077356 | 0.0002 | 5.83 |
| 4 | 177605082 | *VEGFC* | 1258TCA> | S420 | rs5864401 | 0.003 | 2 |
| 6 | 116573958 | *TSPYL4* | 1214C>T | S405N | rs183681428 | 0.0004 | 0.12 |
| 8 | 10465636 | *RP1L1* | 5972C>T | G1991E | NA | 0 | 0.89 |
| 12 | 9246177 | *A2M* | 2126-6_2126-2 | NA | rs3832852 | 0 | 0.88 |
| X | 154158285 | *F8* | 3780G>C | D1260E | rs1800291 | 0 | -0.19 |

MAF, Minor Allele Frequency; Phylop score (< 0, less conserved; 0, neutral; > 0 conserved; a large score indicates high conservation)
