# Supplementary material for: Whole Exome Sequencing in Patients with the Cuticular Drusen Subtype of Age-Related Macular Degeneration
Source: PLoS One. 2016 Mar 23;11(3):e0152047. doi: 10.1371/journal.pone.0152047 (PMC4805164; doi:10.1371/journal.pone.0152047)
Supplement: S11 Table — (DOCX) [file pone.0152047.s011.docx]

**S11 Table. Sporadic case 9AB, Fig 2**

| **Chromosome** | | **Gene** | **Change in** | | **SNP id** | **MAF** | **Conservation** |
| --- | --- | --- | --- | --- | --- | --- | --- |
| **#** | **Position** |  | **Nucleotide** | **Amino acid** |  |  | **Phylop (Base level)** |
| 1 | 196709816 | *CFH* | 2850G>T | Q950H | rs149474608 | 0.002 | -0.74 |
| 2 | 216251580 | *FN1* | 4171G>A | R1391C | rs116558455 | 0.001 | 4.21 |
| 4 | 177605082 | *VEGFC* | 1258TCA> | S420 | rs5864401 | 0.003 | 2 |
| 5 | 127744469 | *FBN2* | 976G>A | P326S | rs28763954 | 0.003 | 2.71 |
| 6 | 30893728 | *VARS2* | 3123C>G | D1041E | NA | 0 | -0.06 |
| 6 | 42153428 | *GUCA1B* | 465C>A | E155D | rs139923590 | 0.003 | 0.66 |
| 12 | 6061684 | *VWF* | 7988C>A | R2663L | NA | 0 | 0.81 |
| 12 | 6101167 | *VWF* | 6616A>G | S2206P | NA | 0 | 0.83 |
| 19 | 14501850 | *CD97* | 305G>A | G102E | NA | 0 | 0.66 |

MAF, Minor Allele Frequency; Phylop score (< 0, less conserved; 0, neutral; > 0 conserved; a large score indicates high conservation)
