# Supplementary material for: Whole Exome Sequencing in Patients with the Cuticular Drusen Subtype of Age-Related Macular Degeneration
Source: PLoS One. 2016 Mar 23;11(3):e0152047. doi: 10.1371/journal.pone.0152047 (PMC4805164; doi:10.1371/journal.pone.0152047)
Supplement: S12 Table — (DOCX) [file pone.0152047.s012.docx]

**S12 Table. Sporadic case 10AB, Fig 2**

| **Chromosome** | | **Gene** | **Change in** | | **SNP id** | **MAF** | **Conservation** |
| --- | --- | --- | --- | --- | --- | --- | --- |
| **#** | **Position** |  | **Nucleotide** | **Amino acid** |  |  | **Phylop (Base level)** |
| 4 | 154626317 | *TLR2* | 2258G>A | R753Q | rs5743708 | 0.007 | 4.54 |
| 4 | 155533035 | *FGG* | 323G>C | A108G | rs148685782 | 0.002 | 1.82 |
| 4 | 177605082 | *VEGFC* | 1258TCA> | S420 | rs5864401 | 0.003 | 2 |
| 6 | 76640670 | *IMPG1* | 2243C>A | R748M | NA | 0 | 3.62 |
| 6 | 30893728 | *VARS2* | 3123C>G | D1041E | NA | 0 | -0.06 |
| 10 | 50680422 | *ERCC6* | 2924C>T | R975Q | rs145720191 | 0.001 | 6.2 |
| 12 | 7177276 | *C1S* | 1388C>T | A463V | rs375077429 | 0 | 2.63 |
| 12 | 43886984 | *ADAMTS20* | 940G>A | H314Y | NA | 0 | 2.05 |
| 14 | 92403294 | *FBLN5* | 376C>T | V126M | rs61734479 | 0.0008 | 3.99 |

MAF, Minor Allele Frequency; Phylop score (< 0, less conserved; 0, neutral; > 0 conserved; a large score indicates high conservation)
