# Supplementary material for: Whole Exome Sequencing in Patients with the Cuticular Drusen Subtype of Age-Related Macular Degeneration
Source: PLoS One. 2016 Mar 23;11(3):e0152047. doi: 10.1371/journal.pone.0152047 (PMC4805164; doi:10.1371/journal.pone.0152047)
Supplement: S13 Table — (DOCX) [file pone.0152047.s013.docx]

**S13 Table. Sporadic case 11AB, Fig 2**

| **Chromosome** | | **Gene** | **Change in** | | **SNP id** | **MAF** | **Conservation** |
| --- | --- | --- | --- | --- | --- | --- | --- |
| **#** | **Position** |  | **Nucleotide** | **Amino acid** |  |  | **Phylop (Base level)** |
| 1 | 57395228 | *C8B* | 1625G>A | T542I | rs61737417 | 0.003 | 0.66 |
| 2 | 21238301 | *APOB* | 3449A>T | M1150K | rs146223051 | 0 | 1.91 |
| 3 | 124483297 | *ITGB5* | 2245G>T | H749N | NA | 0 | 5.94 |
| 4 | 187157943 | *KLKB1* | 337C>T | R113* | rs121964949 | 0 | -0.05 |
| 4 | 177605082 | *VEGFC* | 1258TCA> | S420 | rs5864401 | 0.003 | 2 |
| 6 | 161128812 | *PLG* | 266G>A | R89K | rs143079629 | 0.002 | 1.89 |
| 8 | 17731611 | *FGL1* | 419T>A | Y140F | rs35431851 | 0.007 | 4.66 |
| 10 | 124189183 | *PLEKHA1* | 944C>T | P315L | NA | 0 | 3.48 |
| 12 | 6140659 | *VWF* | 2771C>T | R924Q | rs33978901 | 0.007 | 1.4 |
| 19 | 6759632 | *SH2D3A* | 469G>A | R157W | rs148876828 | 0.0004 | 0.79 |

MAF, Minor Allele Frequency; Phylop score (< 0, less conserved; 0, neutral; > 0 conserved; a large score indicates high conservation)
