# Supplementary material for: Whole Exome Sequencing in Patients with the Cuticular Drusen Subtype of Age-Related Macular Degeneration
Source: PLoS One. 2016 Mar 23;11(3):e0152047. doi: 10.1371/journal.pone.0152047 (PMC4805164; doi:10.1371/journal.pone.0152047)
Supplement: S14 Table — (DOCX) [file pone.0152047.s014.docx]

**S14 Table. Sporadic case 12AB, Fig 2**

| **Chromosome** | | **Gene** | **Change in** | | **SNP id** | **MAF** | **Conservation** |
| --- | --- | --- | --- | --- | --- | --- | --- |
| **#** | **Position** |  | **Nucleotide** | **Amino acid** |  |  | **Phylop (Base level)** |
| 1 | 230845755 | *AGT* | 842T>C | Y281C | rs56073403 | 0.0008 | 1.96 |
| 2 | 56145171 | *EFEMP1* | 146T>G | D49A | rs55849640 | 0.0004 | 2.33 |
| 4 | 177605082 | *VEGFC* | 1258TCA> | S420 | rs5864401 | 0.003 | 2 |
| 6 | 116263644 | *FRK* | 1451C>T | R484H | rs141525046 | 0.001 | 1.85 |
| 8 | 10464616 | *RP1L1* | 6992G>A | T2331M | rs147334256 | 0.0004 | -0.96 |
| 8 | 97620669 | *SDC2* | 413G>C | S138T | NA | 0 | 3.04 |
| 13 | 94197611 | *GPC6* | 256C>T | L86F | rs143872144 | 0.002 | 1.61 |

MAF, Minor Allele Frequency; Phylop score (< 0, less conserved; 0, neutral; > 0 conserved; a large score indicates high conservation)
