# Supplementary material for: Whole Exome Sequencing in Patients with the Cuticular Drusen Subtype of Age-Related Macular Degeneration
Source: PLoS One. 2016 Mar 23;11(3):e0152047. doi: 10.1371/journal.pone.0152047 (PMC4805164; doi:10.1371/journal.pone.0152047)
Supplement: S15 Table — (DOCX) [file pone.0152047.s015.docx]

**S15 Table. Unconfirmed rare sequence variants**

Rare sequence variants not confirmed by Sanger sequencing in cases with CD subtype of AMD

| **Gene** | **Change in** | | **# Family** | **# Sporadic cases** |
| --- | --- | --- | --- | --- |
|  | **Nucleotide** | **Protein** | **(Figure 1)** | **(Figure 2)** |
| *ADAMTS20* | 4742T>G | K1581T | 2 | 0 |
| *ITGA1* | 2758T>G | L920V | 6 | 0 |
| *COL15A1* | 4094A>T | K1365I | 0 | 3AB; 5AB |
|  |  |  |  |  |
